# Acute Compartment Syndrome of the Hand from a Parrot Bite

**DOI:** 10.7759/cureus.3845

**Published:** 2019-01-08

**Authors:** Marc P Dotson, Jeff Mullen

**Affiliations:** 1 Emergency Medicine, Charleston Area Medical Center, Charleston, USA

**Keywords:** compartment syndrome, fasciotomy, zoonosis

## Abstract

Acute compartment syndrome (ACS) of the hand is uncommon, especially secondary to exotic animal bites. In this case, we describe a patient who developed ACS of the hand after being bitten by her pet, an African grey parrot. The patient required emergent fasciotomy that resulted in symptom improvement. Furthermore, the patient developed an abscess leading to the consideration of antibiotic coverage for bird bites. This case provoked the consideration of compartment syndrome on the differential for minor crush or unusual injuries. It is also important to recognize that compartment syndrome may occur in any fascial compartment. As bird-related injuries are uncommon, we have briefly discussed antibiotic coverage for bird bites and associated zoonoses. We believe this is the first reported case of compartment syndrome of the hand from a parrot bite.

## Introduction

Acute compartment syndrome (ACS) can occur in any compartment of the body. While considered uncommon, acute ACS has been reported from various injuries to the hand [1-5]. Exotic animal bites, including birds, are becoming more frequently reported in the literature [6-8]. In this report, we have described the first known case of ACS of the hand from a bird bite. Secondarily, the patient developed an abscess in her forearm hours after the initial injury, prompting discussion of the various micro-pathogens from a bird bite that must be covered.

## Case presentation

A 77-year-old left hand-dominant female first presented to the emergency department (ED) as a transfer from an outlying facility (OLF) secondary to an African grey parrot (*Psittacus erithacus*) bite. The patient was cleaning her pet parrot’s cage at 09:30 hours when she sustained a bite to the dorsum of the right hand. She experienced immediate pain but upon examination, there were no breaks in the skin. The patient then washed the area with antibacterial soap and applied an ice pack. Throughout the day, the pain, swelling, and bruising continued to increase and eventually spread to her dorsal wrist. The pain became unbearable and so she sought care at her local ED. The OLF performed labs and a hand X-ray. She was given one dose of intravenous (IV) ceftriaxone 1 g and transferred to our ED for evaluation by hand surgery. The patient arrived at our facility and was evaluated at 1931. The patient was complaining of increased pain, numbness, and coolness all over the dorsum of the hand. She also stated the swelling had progressed to the distal third of the dorsal forearm from the time of evaluation at the OLF until the time of arrival. She denied systemic symptoms.

The patient’s initial vital signs were temperature 36.9 °C, blood pressure 160/82, heart rate 95 beats per minute, respiratory rate 18 breaths per minute, and oxygen saturation of 97% on room air. On physical exam, the right hand had ecchymosis and soft tissue edema to all five digits as well as the dorsum of the hand with an extension to the distal third of the dorsal forearm. At the proximal third of the dorsal forearm, there was soft tissue swelling, induration, erythema, and fluctuance. No crepitus was noted. There was no swelling beyond the area of erythema. The right hand was cold when compared to the left hand. The digits were held in slight flexion. There was a decreased range of motion with extension and flexion in all five digits as well as wrist extension. There was tenderness to palpation to all five digits, dorsum of the hand, and the dorsal forearm. Radial and brachial pulses were two plus and equal bilaterally. Median, radial and ulnar nerve sensation were intact.

**Figure 1:**
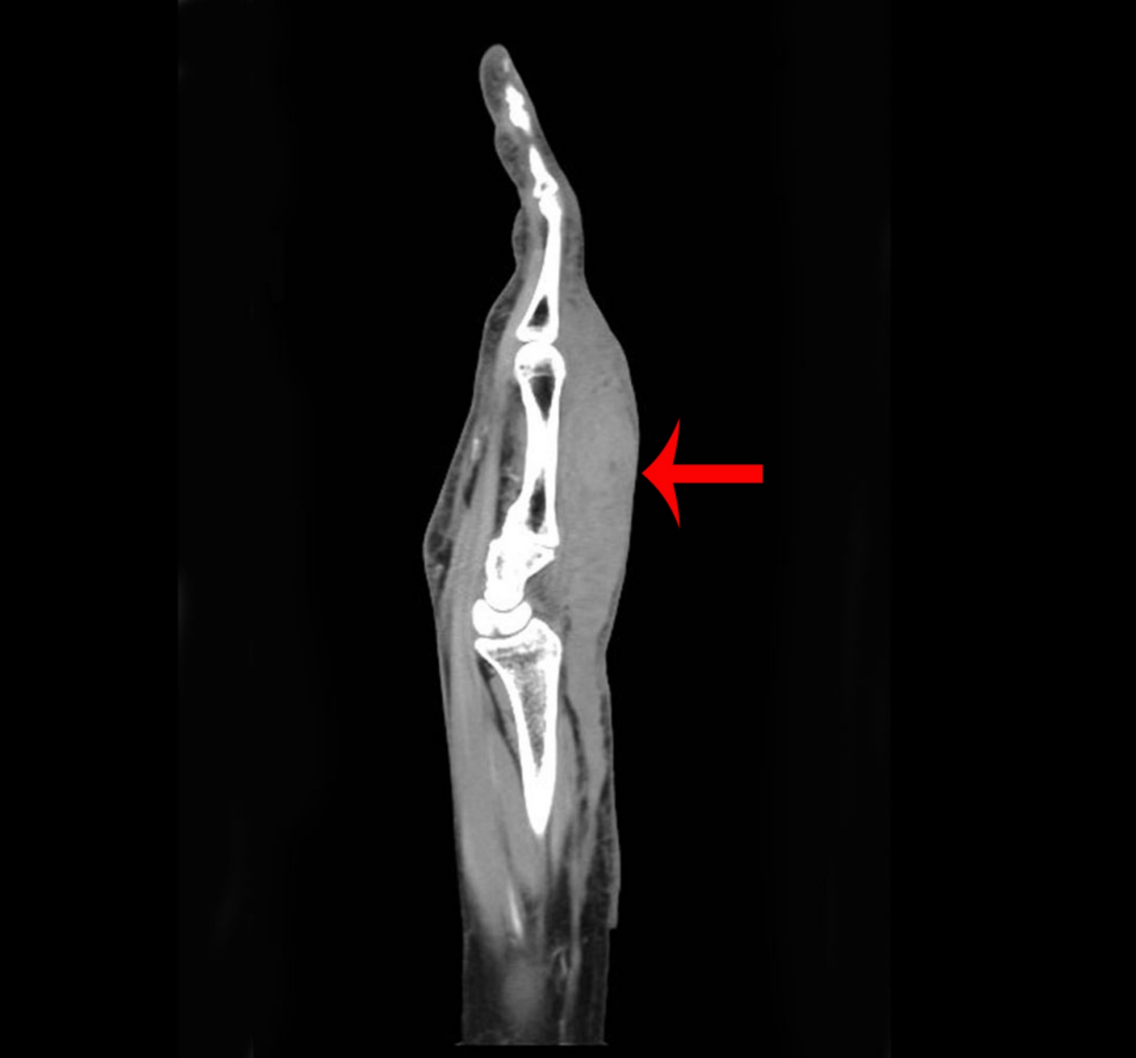
CT scan of hand (coronal view) CT: computed tomography

**Figure 2:**
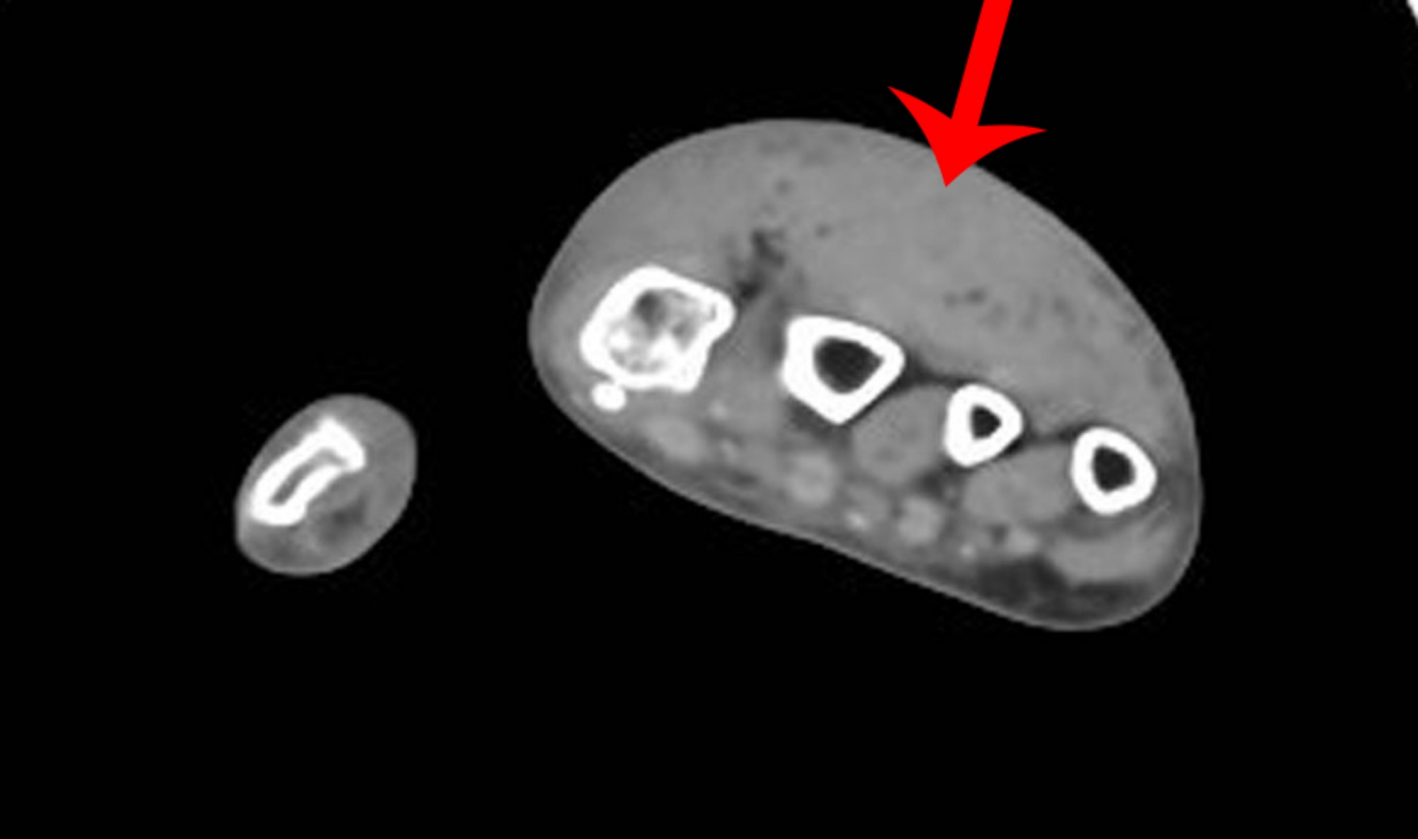
CT scan of the hand (transverse view) CT: computed tomography

The patient had a basic metabolic panel and complete blood count at OLF, which were unremarkable. Her hand X-ray from OLF was interpreted as having soft tissue swelling without evidence of fracture. Given the clinical suspicion of ACS, the hand surgeon was contacted and requested a computed tomography (CT) scan of the extremity with intravenous contrast (Figures 1-2). The patient was evaluated by the hand surgeon at 20:15 hours and taken immediately to the operating room. She was given a tetanus shot and azithromycin 500 mg IV to empirically cover for *C. psittaci* given the history of a bird bite. The CT scan was interpreted as having soft tissue swelling of the dorsal hand without fluid collection or bony abnormality (arrows denote swelling).

In the operative suite, the patient underwent a right-hand fasciotomy. The first incision was made over the third metacarpal into the deep fascia of the dorsum of the hand. A large non-purulent fluid collection was encountered and cultures were obtained. Next, the fascia of the right thenar musculature was released. Finally, the fascia of the right hypothenar musculature was released. After the fasciotomy, the surgeon drained an abscess involving the forearm and iodoform packing was placed. No necrotic tissue was noted. Lastly, the median nerve was released at the carpal tunnel and the ulnar nerve was released at Guyon’s canal to prevent further compression of the nerves. The patient was sent to recovery without complications.

The next day she was evaluated by an infectious disease specialist. They recommended changing her antibiotics to amoxicillin/clavulanate and doxycycline. The patient's wounds continued to improve over the course of her hospitalization. Forty-eight hours after her first procedure, she was taken back to the operative suite for secondary closure of the wounds of the dorsum of the hand and the forearm. All cultures, including aerobic, anaerobic, and fungal, were negative. The patient was discharged home on day three of her admission to complete a 14-day course of antibiotics. She was also given outpatient physical therapy to work on wrist range of motion. At her two-week follow-up, the patient was found to have improved sensation, strength, and range of motion. Her wounds were healing appropriately without any further swelling or drainage.

## Discussion

Domestic animal hand injuries, mostly secondary to scratches or bites, are a common ED complaint. The incidence is reported to be 2 million to 4.7 million, resulting in 300,000 ED visits each year [7,9]. Parrots have the ability to apply forces of 200 pounds per square inch with each bite which can cause significant crush injuries [7,9]. Any type of crush injury, even without fractures, can cause ACS within hours [10].

Our patient developed five out of six clinical signs of compartment syndrome including pain, pallor, paresthesia, paralysis, and poikilothermia. Fasciotomy is the recommended treatment of compartment syndrome involving the hand; however, these recommendations are based off case reports and studies from other fascial compartments [2-3]. The hand comprises eleven compartments with the dorsum being composed of four interossei compartments and aspects of the thenar and hypothenar compartment [1,11]. Interestingly, our patient had a uniform swelling over the entire dorsum of the hand, and only the third interossei, thenar, and hypothenar compartments were released. Involvement of the other dorsal compartments may have been secondary to the skin acting as a constrictor and one midline incision may have been enough to release the pressure exerted on the other compartments.

The patient also developed an abscess that is a reported complication of bird bites [7-9]. The abscess was proximal to the injury site with no extension of purulence to the hand. Additionally, the abscess had developed within hours after the injury. Initially, the patient was given ceftriaxone and then later azithromycin because it is stocked in the ED. While covering for *Staphylococcus* and *Streptococcus* species is important in abscess formation, one must consider other bacteria associated with bites. *Escherichia coli* and zoonoses such as *Pasteurella multocida*, *C. psittaci,* and *Mycobacterium avium* are all considerations when dealing with parrot bites [6-7]. Ceftriaxone will cover *Staphylococcus* and *Streptococcus* species, as well as *E. coli*. Azithromycin will cover *C. psittaci* and *M. avium*. *P. multocida* is covered by third-generation cephalosporins but not macrolides. The patient’s antibiotics were changed to amoxicillin/clavulanate and doxycycline which cover community-acquired bacteria and are the recommended first-line therapies for the aforementioned causes of zoonoses [6-7]. That being said, all cultures were negative and the patient responded to incision and drainage.

## Conclusions

This case illustrates that ACS can occur in any fascial compartment. Although ACS of the hand is rare, it has been well documented in the literature. High clinical suspicion is crucial to make the diagnosis and must be kept on the emergency medicine physician’s differential for any crush type injury. While measuring compartment pressure is useful, it may not be feasible at all facilities and early transfer for evaluation by a surgeon is recommended for a suspected case of ACS. Lastly, this case is a reminder that the emergency medicine physician needs some understanding of empiric antibiotic coverage for rare causes of infection. The global community continues to become smaller and having a differential encompassing non-domestic infectious etiologies will aid patient care.
